# Improving the Intercellular Uptake and Osteogenic Potency of Calcium Phosphate via Nanocomplexation with the RALA Peptide

**DOI:** 10.3390/nano10122442

**Published:** 2020-12-07

**Authors:** Michelle O’Doherty, Eoghan J. Mulholland, Philip Chambers, Sreekanth Pentlavalli, Monika Ziminska, Marine J. Chalanqui, Hannah M. Pauly, Binulal N. Sathy, Tammy H. Donahue, Daniel J. Kelly, Nicholas Dunne, Helen O. McCarthy

**Affiliations:** 1 School of Pharmacy, Queen’s University Belfast, Belfast BT9 7BL, UK; modoherty04@qub.ac.uk (M.O.); emulholland11@qub.ac.uk (E.J.M.); philip.chambers@qub.ac.uk (P.C.); s.pentlavalli@qub.ac.uk (S.P.); m.ziminska@qub.ac.uk (M.Z.); mchalanqui01@qub.ac.uk (M.J.C.); 2 Department of Biomedical Engineering, University Colorado State University, 1374 Campus Delivery, Fort Collins, CO 80523, USA; hmpauly1@gmail.com (H.M.P.); thautdonahue@umass.edu (T.H.D.); 3 Trinity Centre for Bioengineering, Trinity Biomedical Sciences Institute, Trinity College Dublin, Dublin 2, Ireland; binulalns@gmail.com (B.N.S.); kellyd9@tcd.ie (D.J.K.); 4 School of Biomedical Engineering, University of Massachusetts Amherst, 130 Natural Resources Road, Amherst, MA 01003, USA; 5 Department of Mechanical and Manufacturing Engineering, School of Engineering, Trinity College Dublin, Dublin 2, Ireland; 6 Department of Anatomy, Royal College of Surgeons in Ireland, Dublin 2, Ireland; 7 Advanced Materials and Bioengineering Research Centre (AMBER), Royal College of Surgeons in Ireland and Trinity College Dublin, Dublin 2, Ireland; 8 School of Mechanical and Manufacturing Engineering, Dublin City University, Dublin 9, Ireland; 9 Centre for Medical Engineering Research, School of Mechanical and Manufacturing Engineering, Dublin City University, Dublin 9, Ireland; 10 Advanced Manufacturing Research Centre (I-Form), School of Mechanical and Manufacturing Engineering, Dublin City University, Glasnevin, Dublin 9, Ireland; 11 Advanced Processing Technology Research Centre, Dublin City University, Dublin 9, Ireland; 12 School of Chemical Sciences, Dublin City University, Dublin 9, Ireland

**Keywords:** calcium phosphate, peptide, RALA, intercellular, osteogenic, bone engineering

## Abstract

Calcium phosphate-base materials (e.g., alpha tri-calcium phosphate (α–TCP)) have been shown to promote osteogenic differentiation of stem/progenitor cells, enhance osteoblast osteogenic activity and mediate in vivo bone tissue formation. However, variable particle size and hydrophilicity of the calcium phosphate result in an extremely low bioavailability. Therefore, an effective delivery system is required that can encapsulate the calcium phosphate, improve cellular entry and, consequently, elicit a potent osteogenic response in osteoblasts. In this study, collagenous matrix deposition and extracellular matrix mineralization of osteoblast lineage cells were assessed to investigate osteogenesis following intracellular delivery of α-TCP nanoparticles. The nanoparticles were formed via condensation with a novel, cationic 30 mer amphipathic peptide (RALA). Nanoparticles prepared at a mass ratio of 5:1 demonstrated an average particle size of 43 nm with a zeta potential of +26 mV. The average particle size and zeta potential remained stable for up to 28 days at room temperature and across a range of temperatures (4–37 °C). Cell viability decreased 24 h post-transfection following RALA/α-TCP nanoparticle treatment; however, recovery ensued by Day 7. Immunocytochemistry staining for Type I collagen up to Day 21 post-transfection with RALA/α-TCP nanoparticles (NPs) in MG-63 cells exhibited a significant enhancement in collagen expression and deposition compared to an untreated control. Furthermore, in porcine mesenchymal stem cells (pMSCs), there was enhanced mineralization compared to α–TCP alone. Taken together these data demonstrate that internalization of RALA/α-TCP NPs elicits a potent osteogenic response in both MG-63 and pMSCs.

## 1. Introduction

Developments in biomaterial, stem cell and nanomedicine technologies are continually improving the quality of tissue-engineered scaffolds [1,2,3,4,5,6]. However, these applications are limited and do not replicate the innate bone environment [3]. The focus is now on developing strategies aimed at enhancing the intrinsic repair mechanisms of the body to stimulate new, biologically functional bone tissue regrowth to replace tissue that has been lost as a result of trauma or disease [6]. Promoting osteogenic differentiation and bone forming potential of osteoprogenitor/osteoblast cells is an emerging approach for improving bone tissue engineering [7,8,9,10]. Osteoprogenitors and osteoblasts, derived from bone marrow stromal cells, are found at the advancing surface of repairing bone and play a key role in bone production, maintenance and remodeling; producing, depositing and mineralizing the extracellular collagen matrix [11,12]. Therefore, the modulation of the osteogenic properties of these cells represents a potential target to promote soft tissue attachment to bone. Bergemann et al., (2015) have demonstrated the influence of implant material surface topography on the behavior of primary human osteoblasts [13]. This investigation demonstrated that in vitro, micro-structured zirconia surfaces can modulate key bone-related gene expression in human primary osteoblasts, enhancing osseointegration of oral implants. Furthermore, Hu and Olsen (2016) have shown in vitro that osteoblast-derived vascular endothelial growth factor functions as a paracrine factor on osteoblastic lineage cells promoting osteoblastic maturation and mineralization [14].

Calcium phosphate-based materials exhibit similar chemical and structural properties to natural bone minerals and so are widely used in a range of biomedical applications for bone repair: As cements to fill bone defects, coating materials to improve orthopedic implant biocompatibility and in biodegradable bioceramics and composites [15,16,17,18]. Biomaterials containing a calcium phosphate component have been shown to promote osteogenic differentiation of progenitor and stem cells and can facilitate in vivo bone tissue formation [19,20]. In particular, alpha-tricalcium phosphate (α-TCP) has been frequently utilized as a bone substitute material as it is biocompatible, osteoconductive, osteoinductive and biodegradable and elicits no immunogenic effect [21]. Furthermore, implants coated with α-TCP have been shown to improve fixation and integration as the calcium phosphate can form chemical bonds with native bone [22]. Lui et al., (2015) demonstrated that elevated extracellular α-TCP levels enhanced osteogenic differentiation in rat bone mesenchymal stem cells (MSCs) by upregulating a number of osteogenic related-genes [23].

Variable particle size and hydrophilicity of α-TCP limits its bioavailability. Yet the role of intracellular calcium phosphate in the mineralization of the extracellular matrix (ECM) has been demonstrated [24]. Therefore, a more efficacious delivery system is required that can encapsulate the calcium phosphate, improve cellular entry and, consequently, elicit a potent osteogenic response in osteoblasts. To this end, cationic 30 mer amphipathic peptide (RALA) is a novel, non-viral, 30-amino acid peptide gene delivery vehicle, comprised of repeating arginine/alanine/leucine/alanine units [25]. The amphipathic nature of RALA facilitates interaction with the lipid bi-layers, enabling transport across cellular membranes. Furthermore, the pH-sensitive α-helicity of the RALA peptide facilitates endosomal disruption and escape, preventing degradation of the cargo with delivery to the cytosol. Consequently, the RALA delivery system can significantly improve the bioavailability of hydrophilic therapeutics [26,27,28].

In this study, we postulate that the intracellular uptake of α-TCP in the form of nanoparticles (NPs), facilitated by condensation with intracellular delivery peptide RALA, will improve its long-term bioavailability and consequently stimulate the osteogenic potential and bone forming properties of osteoblasts, which could have a key role in bone repair and regeneration.

## 2. Materials and Methods

### 2.1. Materials

#### 2.1.1. RALA Peptide

The RALA peptide was synthesized as previously described [25,26,27,28]. Briefly, the peptide was produced by 9-fluorenylmethyloxycarbonyl (FMOC solid-state peptide synthesis (Biomatik, Washington, DE, USA) and supplied as a desalted, lyophilized powder. The product was purified and validated by reversed-phase high-performance liquid chromatography; molecular mass was confirmed as 3327.98 Da.

#### 2.1.2. Synthesis of Alpha-Tri Calcium Phosphate

The α-TCP (Ca_3_(PO)_4_) powder was synthesized according to the following solid-state reaction [29,30] (Equation (1)):

2CaHPO_4_(s) + CaCO_3_(s) → Ca_3_(PO)_4_(s) + CO_2_(g) + H_2_O(g) (1)

Calcium phosphate (CaHPO_4_) and calcium carbonate (CaCO_3_) precursors were mixed at a molar ratio of 2:1, which was turbo-blended and sintered in a furnace (Elite BRF15/5, Elite Thermal Systems Ltd., Harborough, UK) at 1400 °C for 6 h. The sintered specimens were then removed and placed into a stainless steel bowl and rapidly cooled using compressed air, reducing the possible phase transformation of α-TCP to beta-TCP (β-TCP). The cooled powder was subsequently ground in ethanol (99.5%) using a planetary mill (Pulverisette 6, Frisch, Idar-Oberstein, Germany) at 5 min intervals for a total of 30 min at 600 ± 5 RPM, which produced an average D_10_ particle size of 1.04 ± 0.1 µm, D_50_ of 3.51 ± 0.4 µm and D_90_ of 10.86 ± 0.6 µm as measured by laser diffraction. The milled α-TCP was stored in a desiccator until required.

#### 2.1.3. Plasmid

pEGFP-N1 (GFP) expression plasmid, purchased from Clontech (Mountain View, CA, USA), was amplified in MAX Efficiency^®^ DH5α™ Competent Cells (Life Technologies, Inchinnan, UK), purified using PureLink^®^ HiPure Plasmid Filter Maxiprep Kit (Life Technologies, Inchinnan, UK) and quantified by UV absorption at 260 nm using a NanoDrop 2000c spectrophotometer (Thermo Scientific, Loughborough, UK).

### 2.2. Methods

#### 2.2.1. X-ray Diffraction

Crystalline phase purity was analyzed by X-ray diffraction using an X’Pert PRO MPD (PANalytical Ltd., Malvern, UK) (CuKα radiation, at 40 kV on a rotating stage, Range (2q): 5–6). The JCPDS file from the X’Pert High Score V 2.2b software (PANalytical Ltd., Malvern, UK) was used to identify the α-TCP phase (#09-0348).

#### 2.2.2. Fourier Transform Infra-Red Spectroscopy

Structural information of the α-TCP powder at the molecular level was determined by Fourier transform infrared spectroscopy (FTIR) (FT-IR 4100, Jasco Ltd., Dunmow, UK). For this using KBr pellets of α-TCP powder was prepared and scanned in the wavelength range of 500–4000 cm^−1^ at 256 scans with a resolution of 4 cm^−1^.

#### 2.2.3. Scanning Electron Microscopy

Morphological analysis of the α-TCP powder was performed by scanning electron microscopy (SEM) using the Hitachi Analytical Tabletop Microscope/Benchtop SEM TM3030 system (Hitachi High-Technologies Europe GmbH, Warrington, UK). An acceleration voltage of 5 kV was used. All powders were mounted on aluminum discs and sputter coated with gold under vacuum to induce conductively.

#### 2.2.4. Particle Size and Zeta Potential Analysis

RALA/α-TCP NPs were prepared at mass ratios of 1, 5, 10 and 15 to a final volume of 1 mL; e.g., to achieve a ratio of 10:1, 10 μg of RALA was added to 1 μg of α-TCP in aqueous solution. NPs were allowed to incubate at room temperature for approximately 30 min. In a separate set of experiments, RALA/α-TCP NPs were prepared at a mass ratio of 5 and incubated at: (i) room temperature for 0, 1, 7, 14, 21 and 28 days and (ii) a range of temperatures from 4 to 37 °C. Particle size and zeta potential were subsequently analyzed using the Nano ZS Zetasizer and DTS software (Malvern Instruments, Malvern, UK).

#### 2.2.5. Cell Culture

Human osteosarcoma cells MG-63 (ATCC^®^ CRL-1427™) were purchased from ATCC (Manassas, VA, USA). Cells were maintained and expanded in Eagle’s minimum essential medium (MEM) (Gibco^®^, Life Technologies, Inchinnan, UK) supplemented with 10% fetal bovine serum (FBS) (Life Technologies, Inchinnan, UK) in a humidified incubator at 37 °C and 5% CO_2_. Prior to use, cells were trypsinized, using trypsin/EDTA in phosphate buffered solution (PBS) (2X) (Life Technologies, Inchinnan, UK), centrifuged, and re-suspended in MEM. Osteosarcoma MG-63 cells represent a cell type commonly associated with initial cell material characterization. This cell line is known for the high proliferation potential as they have the ability to continuously divide and grow and are therefore utilized in numerous in vitro biocompatibility studies. However, porcine mesenchymal stem cells (pMSCs) were used to support any conclusions made about biocompatibility and osteogenic functionality of the RALA/α-TCP nanoparticles. pMSCs were isolated from the femora of freshly sacrificed 3–8 month-old pigs, obtained from a slaughterhouse (Agri-Food and Biosciences Institute, Belfast, UK). Bone marrow was harvested and homogenized in Dulbecco’s Modified Eagle Medium (DMEM) + 10% FBS using a 16-gauge needle. Bone marrow suspension was centrifuged at 650 *g* for 5 min. After removing supernatant cells were counted and seeded at a density of 10 × 10^6^ cells/T75 flask in MSC expansion media, DMEM, 1X GlutaMAX, 10% FBS and 1% pen/strep (Gibco^®^, Life Technologies, Inchinnan, UK). Media was replaced every three days and flasks monitored day for emergence of MSCs.

#### 2.2.6. Nanoparticle Transfection

MG-63 cells were seeded in a 96-well flat bottom plate (VWR International, Lutterworth, UK) at a density of 1 × 10^4^ cells per well and incubated in complete culture medium for 24 h. Two hours prior to transfection, the cell culture media was replaced with OptiMEM medium (Invitrogen, Inchinnan, UK) optimized for transfection. The RALA/α-TCP NPs were prepared using a mass ratio of 5 such that the final amount of α-TCP/well was 1 µg, and 5 µg RALA. Untreated wells were also prepared and a blank RALA NP control group (RALA—GFP NPs) such that the amount of RALA was the same as that used for the RALA/α-TCP NPs. Cells were incubated with nanoparticles at 37 °C/5% CO_2_ for 4 h before medium was replaced with complete culture medium. The following day, culture media was replaced with complete medium supplemented with dexamethasone (50 nM), ascorbic acid (50 µg/mL) and β-glycerophate (10 mM). In a separate set of experiments, MG-63 cells were seeded in a 24-well flat bottom plate (VWR International, Lutterworth, UK) at a density of 3 × 10^4^ cells and transfected with RALA/α-TCP NPs prepared using a mass ratio of 5 such that the final amount per well was 6.7 µg, and 33.3 µg for α-TCP and RALA, respectively.

#### 2.2.7. Transmission Electron Microscopy

The RALA/α-TCP NP structure was examined using a transmission electron microscope (TEM, JEOL-100CXII, Welwyn Garden City, UK). NPs were prepared at a mass ratio of 5. A formvar/carbon mesh grid (Agar Scientific, Stansted, UK) was then placed face down on a 10 μL sample of the RALA/α-TCP NPs for 10 min. The grid was dried overnight and stained for 5 min with 5% aqueous uranyl acetate at room temperature.

#### 2.2.8. Cell Viability: MTS Cell Proliferation Assay

MG-63 cell viability post-transfection with RALA/α-TCP NPs was assessed using the CellTiter 96^®^ Aqueous One Solution Cell Proliferation Assay (MTS) (Promega, Southampton, UK) according to the manufacturer’s protocol. At various time points (i.e., 1, 7, 14, 21 and 28 days) post-transfection, 20 µL of CellTiter 96^®^ Aqueous One Solution Reagent was added per well of a 96-well plate containing 100 µL of fresh culture medium and incubated at 37 °C/5% CO_2_ for 2–3 h. The absorbance of each well was measured at 490 nm using microplate reader (PowerWave XS, BIO-TEK^®^, Winooski, VT, USA). Results were expressed as mean relative absorbance (%) compared to the control samples.

#### 2.2.9. Cell Toxicity: Lactate Dehydrogenase Assay

The cytotoxicity of RALA NP transfection was assessed using Pierce lactate dehydrogenase (LDH) cytotoxicity assay kit (Thermo Fisher Scientific, Loughborough, UK) according to manufacturer’s protocol. Briefly, at time points 1, 7, 14, 21 and 28 days post-transfection, 50 µL of media was removed and transferred to a fresh 96-well plate and 50 µL of reaction mixture added. After incubation at room temperature for 30 min, the reaction was stopped and LDH activity was determined by spectrophotometric absorbance at 490 nm and 680 nm (PowerWave XS, BIO-TEK^®^, Winooski, VT, USA). Results were expressed as mean relative absorbance (%) compared to the control samples.

#### 2.2.10. Immunocytochemistry

Transfected and control MG-63 cells were fixed in 4% PFA for 30 min at room temperature and blocked for 1 h in PBS containing 1% bovine serum albumin (BSA, Sigma Aldrich, Gillingham, UK) and 0.01% Tween 20 (Sigma Aldrich, Gillingham, UK). Thereafter cells were incubated for 1 h at room temperature with primary antibodies against collagen (Abcam, Cambridge, UK). After washing in PBS, cells were incubated in appropriate secondary antibodies for 1 h at room temperature and observed under a fluorescence microscope (EVOS FL, Life Technologies, Loughborough, UK). Anti-rabbit and Alexa Fluor 568 IgG (Abcam, Cambridge, UK) was used as secondary antibodies. ImageJ image analysis software (NIH, Bethesda, MD, USA) was subsequently used to quantify the average fluorescence intensity of the cells in each treatment group and made relative to the untreated control cells.

#### 2.2.11. Mineralization: Alizarin Red Stain Assay

The presence of calcium in the cell cultures was determined by Alizarin Red Staining (ARS) (Sigma–Aldrich, UK) at Day 7, 14 and 21 post-transfection with RALA only, RALA/GFP and RALA/α-TCP NPs. Briefly, the pMSCs were rinsed with cold PBS and then fixed in 4% PFA for 30 min. Following a rinse with distilled water, the cells were stained with 40 mM alizarin red (pH 4.2) for 30 min at room temperature.

#### 2.2.12. Alizarin Red CPC Extraction

To quantify the level of matrix mineralization, ARS monolayers were washed in triplicate with dH_2_O. ARS was extracted from the monolayer by incubation in 500 μL 100 mM cetylpyridinium chloride (CPC) for 30 min. The dye was subsequently removed and 200 μL aliquots were transferred to a 96-well plate prior to reading at 540 nm (FLUOstar^®^ Omega BMG LabTech, Aylesbury, UK).

#### 2.2.13. Statistical Analysis

Statistical analysis was performed using GraphPad Prism 6 (GraphPad Software Inc., San Diego, CA, USA). Statistical significance of differences between groups was determined by using one-way ANOVA with the Student-Newman–Keuls post-test. A *p*-value less than 0.05 was considered statistically significant.

## 3. Results

### 3.1. Physiochemical Properties of α-TCP Particles

**Figure 1:**
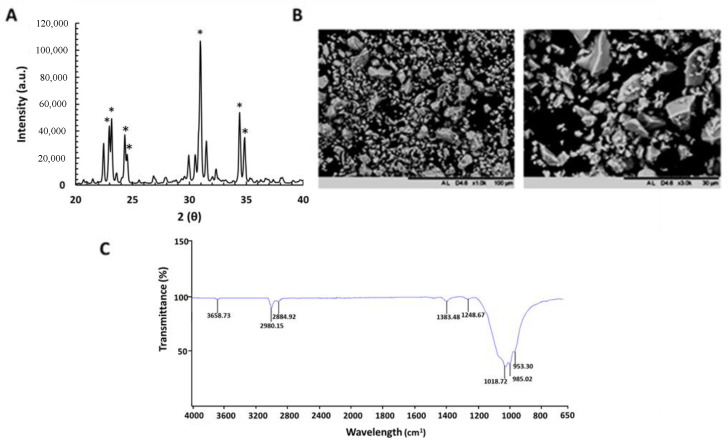
Chemical and morphological characterization of alpha-tricalcium phosphate (α-TCP) powder. (**A**) representative XRD pattern of α-TCP exhibiting characteristic peaks; (**B**) FTIR spectra of α-TCP; and (**C**) SEM micrographs of the surface of α-TCP after final milling step show that the granules of α-TCP have an irregular-shaped morphology. (Scale bar = 100 µm; scale bar = 30 µm), (N = 3 ± SEM).

X-ray diffraction analysis of α-TCP powder showed a peak profile typical of and comparable to α-TCP of a similar standard (JCPDS # 09-0348) (Figure 1A). Characteristic peaks are highlighted by an asterix (*). FTIR analysis confirmed that calcium and phosphate were present in the α-TCP powder (Figure 1B). Formation of tri-calcium phosphate was also confirmed on FTIR analysis as the bands present on α-TCP spectrum (Figure 1C) are characteristic of α-TCP, as previously described [31,32]. SEM showed that the synthesis and milling process produced smooth granules of α-TCP with irregular-shaped morphology (Figure 1B).

### 3.2. Characterization of RALA/α-TCP Nanoparticles

**Figure 2:**
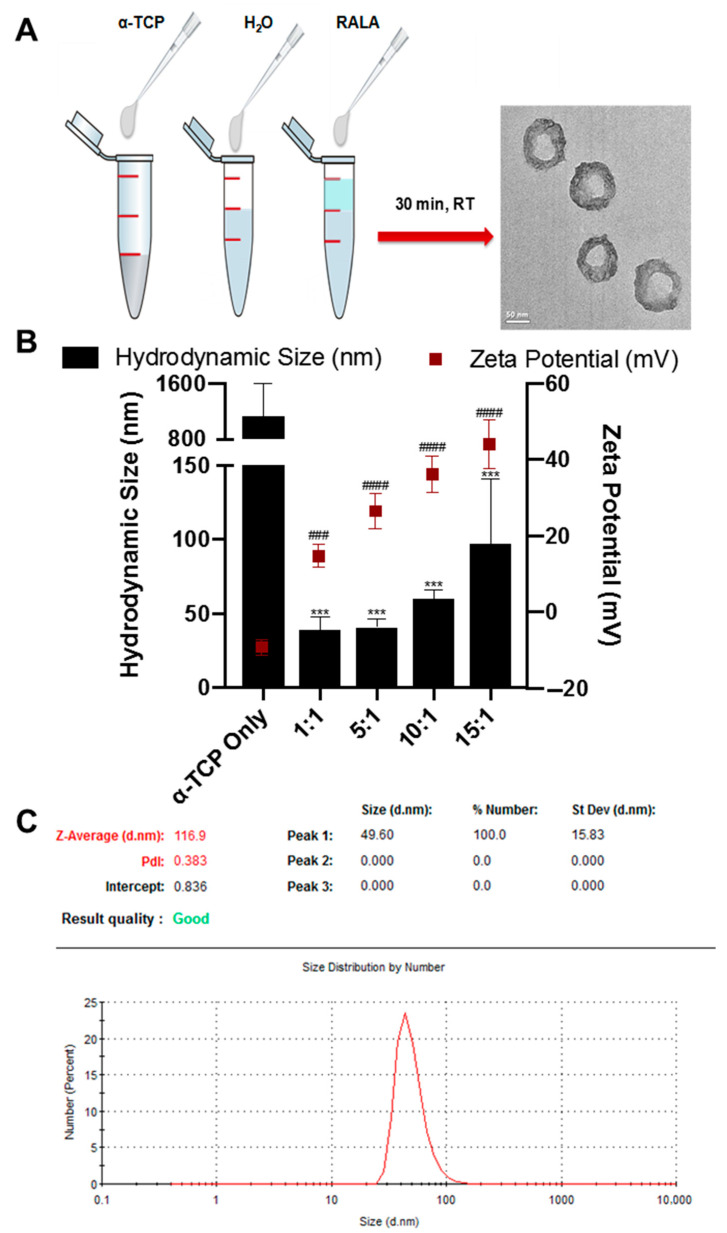
Formulation and characterization of cationic 30 mer amphipathic peptide (RALA)/α-TCP NP. (**A**) RALA/α-TCP nanoparticles (NPs) were prepared by mixing 1 µg α-TCP with reconstituted RALA (lyophilized) at varying mass ratios. Particles were then incubated at room temperature for 30 min. Formulation and morphology of the NPs was confirmed by TEM. (Scale bar = 50 nm). (**B**) Particle size and zeta potential of RALA/α-TCP NPs after formulation at mass ratios (RALA:α-TCP) 1, 5, 10 and 15. The RALA peptide effectively condenses α-TCP into positively charged nanoparticles < 100 nm in size up to mass ratio 15. Significant differences are denoted by the symbols (*) for hydrodynamic size and (#) for zeta potential, compared to α-TCP Only (N = 3 ± SEM) *** = *p* < 0.001, ### = *p* < 0.001, #### = *p* < 0.0001. (**C**) representative size distribution by number of RALA/ α-TCP 7:1.

Effective intracellular uptake is dependent on the size and net charge of the nanoparticle. Therefore, following the formulation of RALA/α-TCP NPs by incubation at room temperature for 30 min (Figure 2A), particle size, charge and shape of RALA/α-TCP were assessed (Figure 2A,B). Results indicated that RALA condensed the α-TCP micro-sized particles into NPs < 100 nm at each mass ratio ranging from 1 to 15. At mass ratio of 5 the average size of the RALA/α-TCP NPs was 43 nm with a zeta potential of +26 mV (Figure 2B). TEM analysis showed that particles exhibited uniform shape and indicated no aggregation (Figure 2A). These biophysical characteristics indicate that the particles are within the ideal size and charge range for receptor mediated endocytosis.

### 3.3. Stability of RALA/α-TCP Nanoparticles

**Figure 3:**
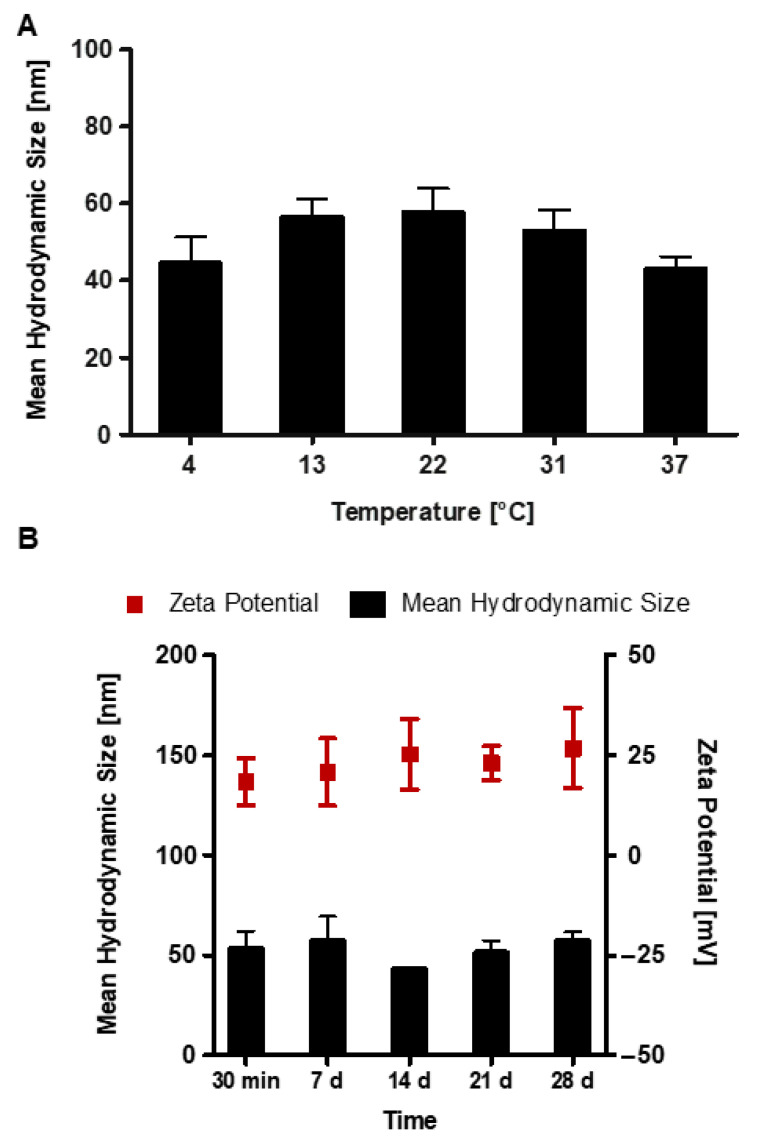
Characterization of RALA/α-TCP NP stability (mass ratio 5). (**A**) particle size remained stable when NPs were incubated over a temperature range of 4–37 °C. (**B**) particle size and zeta potential of RALA/α-TCP NPs were consistent at room temperature over 28 days, i.e., the average particle size was ≈50 nm and ≈+25 mV in charge. (N = 3 ± SEM).

The stability of RALA/α-TCP NP with time and over a temperature range was also investigated (Figure 3). For a mass ratio of 5:1, the particle size remained consistent when exposed to temperatures ranging from 4–37 °C (Figure 3A). NPs were also stable when stored at room temperature over a period of 28 days (Figure 3B), consistently measuring an average particle size of ≈50 nm in size and ≈+25 mV in charge. These results suggest that RALA condensed the α-TCP into stable NPs that retain the ideal characteristics for intercellular delivery with respect to size and charge at 4 °C, room and body temperature.

### 3.4. Cytotoxicity of RALA/α-TCP Nanoparticles

**Figure 4:**
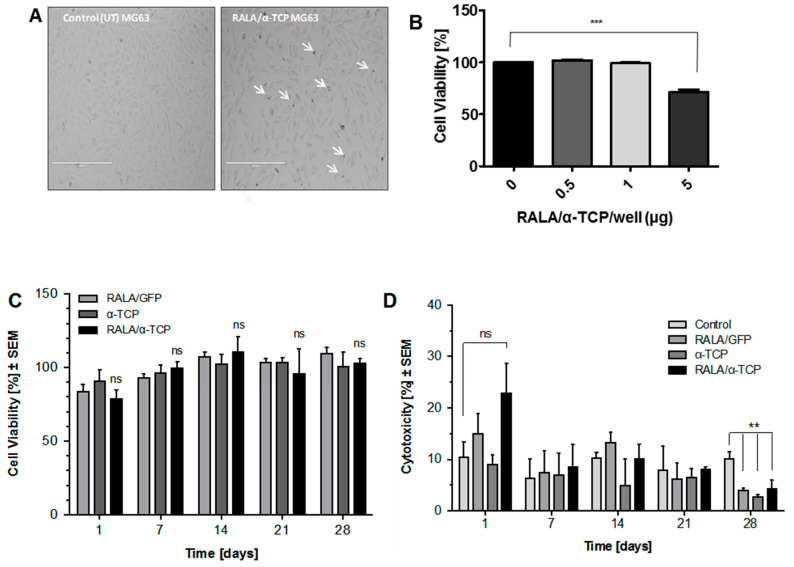
In vitro characterization of RALA/α-TCP transfection and cytotoxicity. (**A**) representative phase contrast images of MG-63 osteoblast-like cells 24 h after transfection with 1 μg RALA/α-TCP NPs at formulation mass ratio 5. White arrows indicate NP deposits. (Scale bar = 400 µm). (**B**) percentage cell viability of MG-63 cells when transfected with varying concentrations of RALA/α-TCP NPs (mass ratio 5); equivalent to 0.5, 1 and 5 g α-TCP/well of 96-well plate. (**C**) and (**D**) percentage cell viability of MG-63 cells over 28 d period after transfection with RALA/α-TCP NPs (equivalent of 1 μg α-TCP/well (96-well plate), mass ratio 5) assessed by (**C**) CellTiter 96^®^ Aqueous One Solution Cell Proliferation Assay (MTS) assay and (**D**) lactate dehydrogenase (LDH) assay. (N = 3 ± SEM). ns = not significant *p* > 0.05, ** = *p* < 0.01, *** = *p* < 0.001.

The transfection concentration and cytotoxic effects of the NPs was assessed in vitro using MG-63 cells, a pre-osteoblast-like-cell (Figure 4A–D). At 24 h post-transfection (addition of RALA/α-TCP NPs so that 1 µg of α-TCP was added to each well of a 96-well plate) cell morphology was observed (Figure 4A). No significant morphological changes were found between the RALA/α-TCP treated cells and the control untreated cells. Dark deposits of RALA/αTCP NPs were observed within the cultures and were not removed upon washing with PBS, suggesting these particles were associated with the cells either intra- or extracellularly. In a separate set of experiments, cells were then transfected with varying amounts of RALA/α-TCP NPs so that 0.5, 1.0 and 5.0 µg of α-TCP were added per well of 96-well plate. Results from the MTS assay indicated that the viability of cells transfected with RALA/α-TCP NP containing 5.0 µg of α-TCP decreased to 71% after 24 h (Figure 4B). Cells treated with NPs containing 0.5 and 1.0 µg α-TCP exhibited comparable cell viability to control untreated cells. Cell viability was then assessed over a longer period via MTS (Figure 4C) and LDH assays (Figure 4D) comparing RALA/α-TCP NP (with α-TCP content 1 µg/well 96-well plate) with RALA/GFP (control NP group with non-osteogenic gene content), free α-TCP supplemented culture media and control untreated cells. Despite an initial increase in cytotoxicity, the 24 h post-transfection (Figure 4D) cell viability of RALA/α-TCP NP treated cells returned to control levels by Day 7. This was supported by a similar finding using the MTS assay (Figure 4C).

### 3.5. Effects of RALA/α-TCP Nanoparticles on Collagen Production in MG-63 Cells

**Figure 5:**
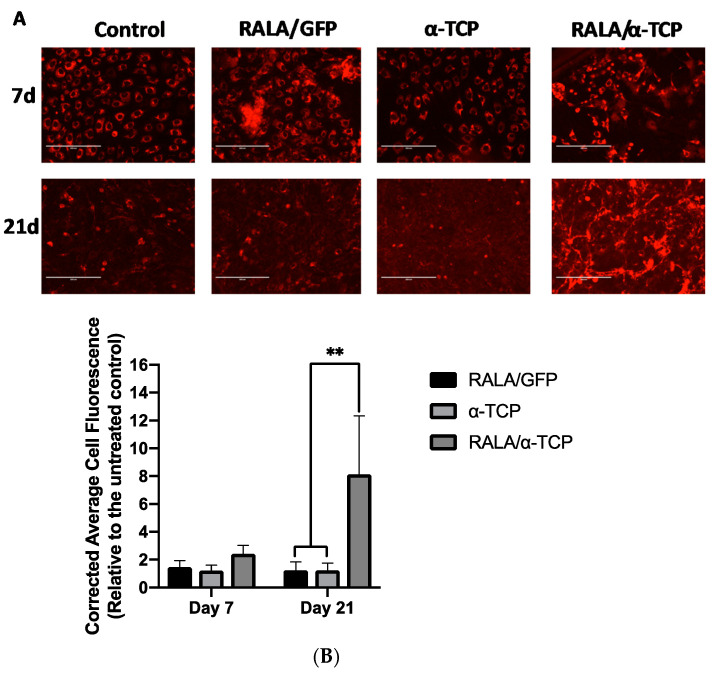
Immunocytochemical analysis for the expression of osteogenic biomarker protein Type I collagen. (**A**) representative fluorescence micrographs of Type I collagen immunofluorescence of MG-63 cell cultures 7 and 21 d post-transfection with RALA/α-TCP NPs in a 24-well plate, evident from the red color. Untreated (control), RALA/pEGFP-N1 (GFP) and α-TCP only groups were also included. (Scale bar = 200 µm). (**B**) quantitative analysis of cell fluorescence intensity as analyzed using ImageJ and results normalized to the untreated (N = 3 ± SEM) ** = *p* < 0.01.

Early osteoblasts deposit large amounts of unmineralized, collagenous extracellular matrix as they differentiate into mature osteoblasts. To determine whether RALA/α-TCP NPs promote collagen production and extracellular deposition, immunocytochemistry staining for Type I collagen was performed to visualize the development of the collagenous matrix surrounding the cells (Figure 5A). Immunostaining confirmed Type I collagen expression for all treatment groups (control, RALA/GFP, α-TCP and RALAα-TCP) from Day 7 up to Day 21 (Figure 5A). However, by Day 21, the level of collagen deposition by RALA/α-TCP NP transfected MG-63 cells were significantly more compared to the other groups (Figure 5B). This result suggests that RALA/α-TCP NPs promote early-stage differentiation of osteoblasts into mature osteoblasts by enhancing collagen matrix deposition.

### 3.6. Effects of RALA/α-TCP Nanoparticles on Calcium Deposition and Matrix Mineralization in pMSCs

**Figure 6:**
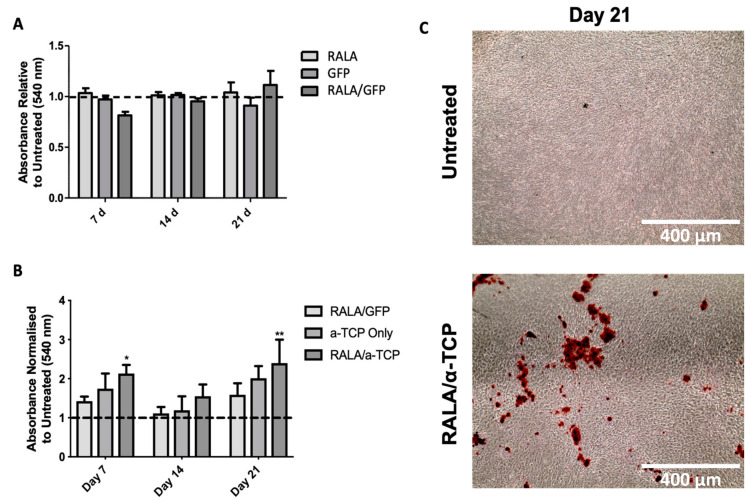
Mineralization of porcine mesenchymal stem cells (pMSC) extracellular matrix. (**A**) cetylpyridinium chloride (CPC) extraction and quantification of mineralized matrix of alizarin red stained pMSC extracellular matrix after transfection with RALA only, GFP only and RALA/GFP NPs in a 24-well plate. (**B**) CPC extraction and quantification of mineralized matrix of alizarin red stained pMSC extracellular matrix after transfection with RALA/GFP, α-TCP only and RALA/ α-TCP in a 24-well plate. (**C**) representative images of untreated and RALA/α-TCP treated pMSC 21 days after transfection (magnification 4×). (N = 3 ± SEM) * = *p* < 0.05, ** = *p* < 0.01.

To evaluate the extent of matrix mineralization as a result of RALA/α-TCP uptake, Alizarin Red Staining was performed to directly compare the extracellular calcium deposits in control, RALA only, RALA/GFP and α-TCP only treated groups along with RALA/α-TCP NP transfected cells up to Day 21 (Figure 6). Porcine MSCs were cultured with RALA only, GFP only and RALA/GFP NPs (Figure 6A). Calcium deposits by RALA/α-TCP transfected cells were first observed at Day 7 (Figure 6B, * *p* < 0.05). The level of mineralized matrix nodules observed in RALA/α-TCP treated cells increased considerably from Day 7 onwards compared to control, and α-TCP only groups. No significant matrix mineralization was observed in the α-TCP only group and limited calcium deposition in the control group was observed at Day 21. However, there was a significant increase in the RALA/ α-TCP group (Figure 6B, ** *p* < 0.01). The results indicate that RALA/α-TCP NPs can accelerate MSC mineralization of collagenous ECM, a key late-stage marker of osteoblast differentiation.

## 4. Discussion

The present study was designed to assess the formulation and biological behavior of RALA/α-TCP NPs to promote osteogenesis and matrix mineralization. We report here for the first time that nanocomplexation of α-TCP by the RALA peptide produces stable, cationic NPs with ideal characteristics for cellular uptake that elicit an osteogenic effect when internalized by pre-osteoblast-like cells. Numerous studies have shown that biomaterials incorporating calcium phosphate, such as bioactive glasses, ceramics, and mineralized matrices, promote bone repair [17,18,19,20,21]. These reports focus on extracellular calcium phosphate-mediated osteoinduction; particularly the effects of topography, microstructure and porosity on osteogenic function [33]. Calcium phosphates exhibit a strong adsorption affinity for endogenous bone growth factors with surface morphology characteristics that facilitate entrapment of these factors within the material [34,35]. Consequently, osteogenic differentiation cannot be fully attributed to the material itself, but to the capacity of the material to adsorb soluble, osteogenic factors at the site of repair. Further studies have subsequently shown that calcium phosphate-based materials with high dissolution rates can induce osteogenesis irrespective of surface geometry and in the absence of soluble osteogenic factors [36]. The dissolution of these materials results in an increase of calcium and phosphate ions. Recently, Shih et al., (2014) demonstrated that uptake of extracellular phosphate by solute carrier family 20 (phosphate transporter), member 1 (SLC20a1), induced osteogenic differentiation of human MSCs via autocrine/paracrine adenosine signaling through the A2b adenosine receptor [19]. This suggests the release and cellular uptake of exogenous calcium and phosphate ions from these calcium phosphate-based materials is the source of their bioactivity, and that calcium phosphate plays a key role in mediating differentiation at the molecular level. However, few have reported the efficacy of α-TCP mediated osteogenic differentiation by direct, intracellular delivery. Bioavailability of α-TCP is limited, and intracellular influx of exogenous ions is tightly regulated to maintain cell integrity therefore, delivery of α-TCP directly to the cytoplasm bypasses a number of limiting biological processes. This study demonstrates that intracellular delivery of α-TCP nanocomplexed with RALA peptide enhances pre-osteoblast-like-cell mineralization compared to cells exposed to exogenous α-TCP supplemented in culture media in terms of the rate and density of mineralization observed. This demonstrates the inherent advantage of RALA/α-TCP NP mediated osteogenic differentiation compared to the more common “outside-in” mediated strategies.

Intracellular drug delivery can significantly increase the efficiency of therapeutic protocols and the efficacy of the treatment [37]. In this study, we demonstrate the charge neutralization and condensation of α-TCP by RALA (Figure 2B) facilitates transport across the cell membrane (Figure 4A) via clathrin endocytosis [25]. Previous studies have shown that the intracellular uptake pathway of fluorescent cationic and anionic calcium phosphate NPs by HeLa cells is mainly via macropinocytosis, leading to localization first in endosomes and then in lysosomes [19]. The pH sensitive α-helicity of RALA facilitates release of the α-TCP from endosomes after endocytosis of the NPs. This protects the α-TCP from degradation by the endosomal pathway, enabling delivery of the calcium phosphate to the cytoplasm.

Biosafety and biocompatibility of any novel pharmaceutical therapeutic are key concerns and necessary to establish early on in the development process [38,39]. MG-63 cells transfected with these NPs showed minimal cytotoxic effects up to Day 28 post-transfection (Figure 4C,D), although concentration is a key factor that with MG-63 cell viability decreased when transfected with 25 µg RALA/ 5µg α-TCP NP per well of 96-well plate (Figure 4B). Very few reports are available on the cytotoxicity associated with calcium phosphate-based NPs and particularly α-TCP NPs. A number of studies have shown that hydroxyapatite NPs induce apoptosis in bone and cartilage cell [40,41,42,43]. Moreover, size, morphology and concentration of hydroxyapatite NPs have all been shown to play a key role in cell apoptosis [44]. Cytotoxic effects of β-TCP NPs on human fetal osteoblast cells have also been linked to the nano-scale size of the β-TCP [45]. In addition, vascular smooth muscle cells exposed to exogenous calcium phosphate crystals exhibit a dramatic rise in intracellular Ca^2+^ ions and subsequent cell death, as a result of plasma membrane damage and cellular uptake of the calcium phosphate NPs [46]. The proposed mechanism for this cytotoxic effect suggests elevated and sustained cytosolic calcium ion concentration, after lysosomal processing of the calcium phosphate crystals, leads to loss of function of membrane pumps leading to cell death [47].

In this study, the initial increase in cytotoxicity after 24 h can be explained on the basis of pH change of the culture medium, plasma membrane damage upon initial NP uptake or exhaustion of membrane pumps due to rapid increase in cytosolic calcium ion concentration. Taken together with our data showing increased mineralization and a general trend of increased osteogenic gene expression, it suggests the α-TCP NPs are well tolerated by the cells long-term, as the viable cells are still fully functional and responsive to the NPs.

There was a significant increase in collagen deposition, detected at the protein level by immunocytochemistry (Figure 5). Type 1 collagen is an early-stage marker of pre-osteoblast differentiation into mature osteoblast [47]. This organic matrix confers elasticity and flexibility to bone tissue [48]. The gene expression studies (Figure S1) while not statistically significant showed a general trend of increased RUNX2 and Alkaline phosphatase expression in MG-63 treated with α-TCP NPs at day 21. The immunocytochemistry findings further suggest that the NP treated MG-63 cells are differentiating and progressing towards a mature osteoblast phenotype. This implies that the RALA/α-TCP NP can modulate osteogenic differentiation and promote bone tissue formation.

Mineralization of bone ECM provides mechanical strength and rigidity to bone [49]. RALA/α-TCP NPs induced mineralization in MSC cultures more rapidly and to a greater extent than untreated control and α-TCP only groups (Figure 6). This suggests that elevated intercellular calcium and phosphate levels mediate matrix mineralization in pMSCs. Both calcium and phosphate ions promote the activity of osteoblasts-lineage cells [50]. Raised calcium concentrations inside and outside the cell have been implicated in matrix mineralization [24,51]. Indeed, ECM mineralization has previously been linked to intracellular phosphate uptake via adenosine signaling [19]. Therefore, our results suggest that by increasing the intracellular bioavailability of calcium and phosphate ions via RALA/α-TCP NP delivery, ECM mineralization is stimulated compared to a greater extent compared to untreated and α-TCP only supplemented culture media. Recently, it has been suggested that amorphous calcium phosphate and ionic calcium stored in the mitochondria are transported via vesicles to the ECM where crystalline apatite propagates from dense foci [24]. A similar process may be proposed for the fate of the intracellularly delivered α-TCP. However, once delivered to the cytosol the fate of the α-TCP is still relatively unknown. Further studies to examine the intracellular fate of the α-TCP and elucidate the mechanisms involved in the RALA/α-TCP mediated matrix mineralization are, therefore, required.

## 5. Conclusions

We have demonstrated that RALA condenses α-TCP into nanoparticles when prepared at mass ratios of 5:1 to 15:1. For a mass ratio of 5:1, the RALA/α-TCP NPs exhibited an average particle size of 43 nm and charge of +26 mV, which are ideal characteristics for cellular entry. The average particle size and zeta potential remained stable for up to 28 days at room temperature and across a range of temperatures (4–37 °C). Furthermore, these NPs are stable, non-toxic and capable of increasing osteogenesis in both MG-63 and pMSCs. The nanoparticles exhibited significant increases in collagen deposition as well as enhanced mineralization potential. Further differentiation studies are required to characterize and validate the osteogenic potential of our RALA/α-TCP NPs in MSCs. The results to date indicate that the RALA/α-TCP NP system has potential as an effective and safe treatment strategy for enhancing osteogenesis.

## Supplementary Materials

The following are available online at https://www.mdpi.com/2079-4991/10/12/2442/s1, Figure S1: Osteogenesis-related gene expression by real-time PCR.
