# Supplementary material for: Improving the Intercellular Uptake and Osteogenic Potency of Calcium Phosphate via Nanocomplexation with the RALA Peptide
Source: Nanomaterials (Basel). 2020 Dec 7;10(12):2442. doi: 10.3390/nano10122442 (PMC7762210; doi:10.3390/nano10122442)
Supplement: Supplementary file 1 [file nanomaterials-10-02442-s001.pdf]

nanomaterials

Supplementary Materials

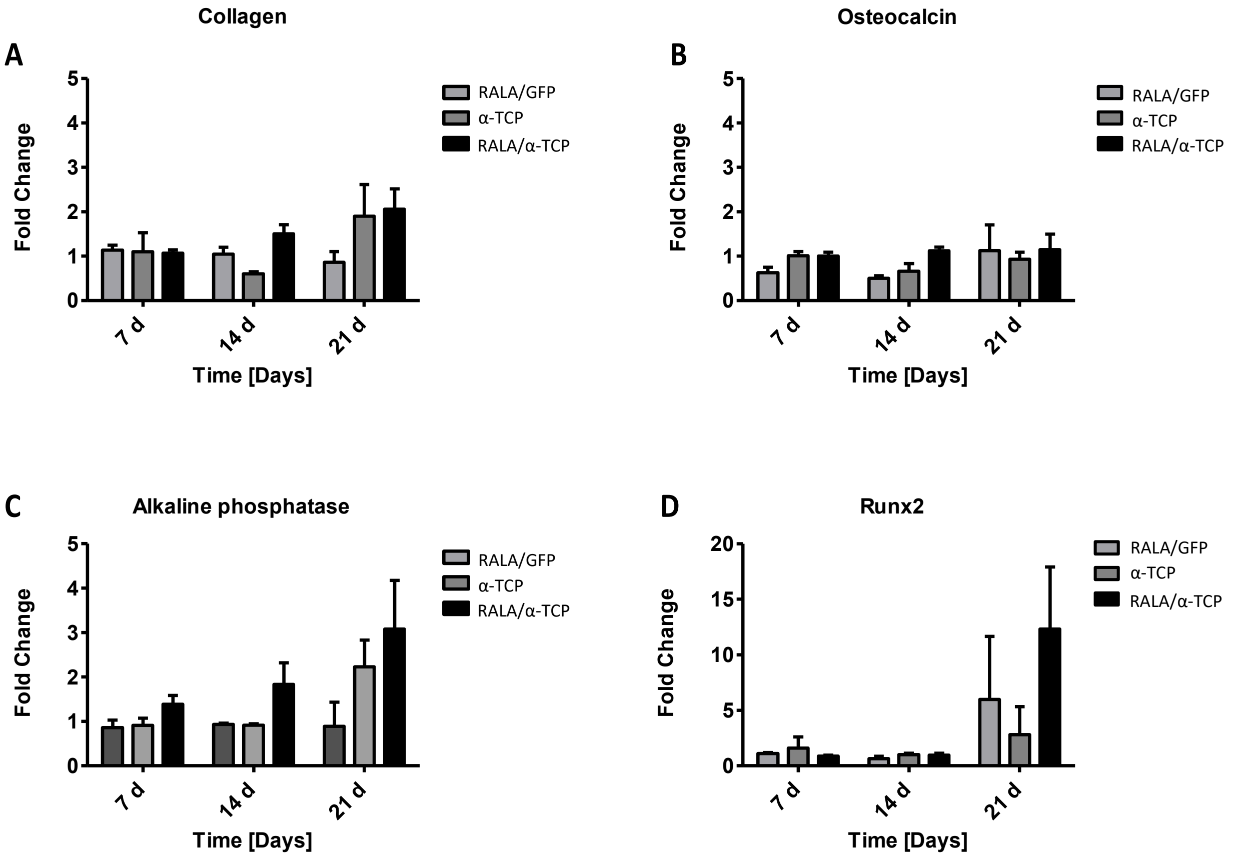

Supplementary Figure 1. Osteogenesis-related gene expression by real-time PCR. The average mRNA levels of the RT-PCR products of osteogenic genes: collagen I, osteocalcin, alkaline phosphatase and Runx2, expressed by MG-63 cells cultured up to 21 d post-transfection with RALA/α-TCP NPs in a 24-well plate. Expression was normalised to untreated control MG-63 cells, using GAPDH as the reference, and the relative gene expression was determined by using REST software (Qiagen, UK). (N = 3 ± SEM). Control vs RALA/α-TCP $p>0.05$ for all genes, across all time points.
